# Identification alone versus intraoperative neuromonitoring of the recurrent laryngeal nerve during thyroid surgery: experience of 2034 consecutive patients

**DOI:** 10.1186/1916-0216-43-16

**Published:** 2014-06-18

**Authors:** Pietro Giorgio Calò, Giuseppe Pisano, Fabio Medas, Maria Rita Pittau, Luca Gordini, Roberto Demontis, Angelo Nicolosi

**Affiliations:** 1 Department of Surgical Sciences, University of Cagliari, Cagliari, Italy; 2 School of Specialty in Forensic Medicine, University of Cagliari, Cagliari, Italy; 3 Department of Public Health, Clinical and Molecular Medicine, University of Cagliari, Cagliari, Italy

**Keywords:** Neuromonitoring, Recurrent laryngeal nerve, Thyroidectomy

## Abstract

**Background:**

The aim of this study was to evaluate the ability of intraoperative neuromonitoring in reducing the postoperative recurrent laryngeal nerve palsy rate by a comparison between patients submitted to thyroidectomy with intraoperative neuromonitoring and with routine identification alone.

**Methods:**

Between June 2007 and December 2012, 2034 consecutive patients underwent thyroidectomy by a single surgical team. We compared patients who have had neuromonitoring and patients who have undergone surgery with nerve visualization alone. Patients in which neuromonitoring was not utilized (Group A) were 993, patients in which was utilized (group B) were 1041.

**Results:**

In group A 28 recurrent laryngeal nerve injuries were observed (2.82%), 21 (2.11%) transient and 7 (0.7%) permanent. In group B 23 recurrent laryngeal nerve injuries were observed (2.21%), in 17 cases (1.63%) transient and in 6 (0.58%) permanent. Differences were not statistically significative.

**Conclusions:**

Visual nerve identification remains the gold standard of recurrent laryngeal nerve management in thyroid surgery. Neuromonitoring helps to identify the nerve, in particular in difficult cases, but it did not decrease nerve injuries compared with visualization alone. Future studies are warranted to evaluate the benefit of intraoperative neuromonitoring in thyroidectomy, especially in conditions in which the recurrent nerve is at high risk of injury.

## Background

Operation on the thyroid gland are the most frequently performed endocrine procedures worldwide. Improvements in technique have decreased the risk of injury to adjacent structures to minimal levels [1]. Apart from hypoparathyroidism [2,3] and hematoma [3,4], recurrent laryngeal nerve palsy is the most common and serious complication after thyroid surgery [2,3,5,6] and it is the leading cause of medicolegal litigation after endocrine surgery [2,3,7].

The rate of recurrent laryngeal nerve palsy varies from 0.5 to 20% depending on the type of disease (benign or malignant), the type of surgery (first time or reoperation), the extent of resection, and the surgical technique [5,6,8-14]; abnormal anatomy, bulky disease and surgeon inexperience are additional risk factors of recurrent laryngeal nerve injury [3,6,8,15-17].

Recurrent laryngeal nerve injury can diminish quality of life due to a variety of symptoms related to voice changes and subsequent limitations in physical, emotional and social functioning, while bilateral injury can be a life-threatening complication leading to airway obstruction [6,18,19].

Intraoperative identification of the recurrent laryngeal nerve has been demonstrated to decrease the incidence of postoperative nerve palsy [5-7,15,18,20,21]. But even in the most experienced hands recurrent laryngeal nerve palsy occurs occasionally, with permanent palsy rate of 1-2% and temporary palsy rate of up to 5-6%, owing to variability in nerve anatomy and difficulties in nerve identification [2,5,15,22-26].

The causes of recurrent laryngeal nerve injury could result from transaction, clamping, stretching, electro-thermal injury, ligature entrapment, or ischemia [24,27-30]; several studies have reported that anatomical variations of the recurrent laryngeal nerve, such as extralaryngeal branches, distorted recurrent laryngeal nerve, intertwining between branches of the recurrent laryngeal nerve and inferior thyroid artery and non-recurrent laryngeal nerve, play an important role in the occurrence of nerve injury that can be caused by visual misidentification [24].

In recent years, increasing attention has been paid to the use of neuromonitoring devices to reduce the risk of nerve injury during thyroid surgery [15,31]. Many studies reported a high negative predictive value of 92-100% but a low and highly variable positive predictive value of 10-90% [2,32]. Recurrent laryngeal nerve monitoring is being used with increasing frequency in the United States during thyroidectomy, partly driven by the medico-legal system [10,22,33-35] while German surgeons use the method routinely even much more often than the Americans [1]; the rates of the use of neuromonitoring have recently become almost equivalent between general surgery- and otolaryngology-trained surgeons [14,22,36]. However, the use of recurrent laryngeal nerve monitoring is associated with increased time of setup and increased cost of equipment by about 5-7% [1,33]. The economic impact is considerable, owing to the costs of the technology, which, for the entire equipment set-up, could vary from 15,000 to 20,000 Euro [25,37].

The aim of the present retrospective study was to evaluate the ability of this technique to predict the postoperative functional outcome and the role of intraoperative neuromonitoring in reducing the postoperative recurrent laryngeal nerve palsy rate by a comparison between patients submitted to thyroidectomy with intraoperative neuromonitoring and with routine identification alone in a single endocrine surgical centre.

## Methods

Between June 2007 and December 2012, 2034 consecutive patients underwent thyroidectomy by a single surgical team. 1749 patients were submitted to total thyroidectomy, 231 to total thyroidectomy with VI level lymphectomy, and 54 underwent completion total thyroidectomy. 1615 were female and 419 male with a mean age of 51 years (range 15–87 years).

The final diagnosis were: in 751 cases multinodular goiter (36.9%), in 615 differentiated carcinoma (30.2%), in 424 Hashimoto’s thyroiditis (20.8%), in 215 Graves’ disease (10.6%), in 29 medullary carcinoma (1.4%).

Histological diagnosis and surgical procedures are summarized in Table 1.

**Table 1:** Histological diagnosis and surgical procedures

	**Total n (%)**	**Total thyroidectomy n (%)**	**Completion thyroidectomy n (%)**	**Total thyroidectomy + lymphectomy n (%)**
Group A	993 (100%)	854 (86%)	40 (4.03%)	99 (9.97%)
• Multinodular goiter	374 (37.66%)	337 (90.11%)	20 (5.35%)	17 (4.55%)
• Differentiated carcinoma	285 (28.7%)	212 (74.39%)	7 (2.46%)	66 (23.16%)
• Hashimoto’s thyroiditis	216 (21.75%)	195 (90.28%)	12 (5.56%)	9 (4.17%)
• Graves’ disease	104 (10.47%)	101 (97.12%)	1 (0.96%)	2 (1.92%)
• Medullary carcinoma	14 (1.41%)	9 (64.29%)	0	5 (35.71%)
Group B	1041 (100%)	895 (85.98%)	14 (1.34%)	132 (12.68%)
• Multinodular goiter	377 (36.22%)	341 (90.45%)	7 (1.86%)	29 (7.69%)
• Differentiated carcinoma	330 (31.7%)	238 (72.12%)	4 (1.21%)	88 (26.67%)
• Hashimoto’s thyroiditis	208 (19.98%)	199 (95.67%)	2 (0.96%)	7 (3.37%)
• Graves’ disease	111 (10.66%)	106 (95.5%)	1 (0.9%)	4 (3.6%)
• Medullary carcinoma	15 (1.44%)	11 (73.33%)	0	4 (26.67%)

In differentiated carcinoma, lymph node metastasis were found in 49 patients (7.6%) and micrometastasis in 14 (2.21%). Totally lymph node metastasis were observed in 61 patients (9.47%).

The study was approved by the Bioethics Commitee of the University of Cagliari. All patients provided written informed consent for the intervention and for the storage and use of their data.

All operations were performed by three experienced endocrine surgeons, with a standard Kocher’s incision. All patients were submitted to preoperative and postoperative laryngoscopy. Post-operative laryngoscopy was performed 2 days after the surgical procedure.

The recurrent laryngeal nerves were routinely identified by visualization and completely exposed. In 1711/2034 operations the relation between nerves and inferior thyroid artery and its branches was described.

Recurrent laryngeal nerves were posterior to inferior thyroid artery in 85.44% of cases on the right side and 82.81% on the left. Nerves were anterior in 5.4% of cases on the right and 5.01% of cases on the left. Nerves with extralaryngeal bifurcation were 2.38% on the right and 2.3% on the left, whilst course of the nerve between the branches of the artery was found in 5.78% on the right and 5.21% on the left. In 1% of cases a non recurrent nerve or a nerve with an extralaryngeal trifurcation was found.

Intraoperative neuromonitoring was performed in 1041 patients on the basis of the availability of the equipment (2068 nerves at risk). All these patients undergone general anesthesia and were intubated with Nerve Integrity Monitor Standard Reinforced Electromiography Endotracheal Tube (Medtronic Xomed). The tube was placed with the middle of the blue-marked region (3 cm of the exposed electrodes) well in contact with the true vocal cords under direct laryngoscopy. When the monitor was well set up, we routinely checked the impedance of electrodes. A Prass monopolar stimulation probe (Medtronic Xomed) was used for nerve stimulation during thyroidectomy. EMG activity was recorded on a NIM-response 2.0 or 3.0 monitor (Medtronic Xomed). No muscle relaxants were used after the skin flaps were elevated.

The neuromonitoring device was used in various phases of the operation: at the beginning, a stimulation was done to the level corresponding to the vagus nerve to ensure that the monitoring system was working; after, to the structure believed to be the inferior laryngeal nerve; at the end, to the level of both the vagus and the recurrent nerve after the removal of thyroid and the hemostasis of the surgical field for predicting the postoperative outcome.

Patients in which the nerve monitoring did not function properly were excluded from the study.

We compared patients who have had neuromonitoring and patients who have undergone surgery with nerve visualization alone.

Patients in which neuromonitoring was not utilized (Group A) were 993 (1946 nerves at risk), 169 male and 824 female. 854 (86%) were submitted to total thyroidectomy, 40 (4.03%) to completion total thyroidectomy and 99 (9.97%) to a total thyroidectomy associated to a VI level lymphectomy. In this group diagnosis was benign multinodular goiter in 374 (37.66%) patients, differentiated carcinoma in 285 (28.7%), Hashimoto’s thyroiditis in 216 (21.75%), Graves’ disease in 104 (10.47%), medullary carcinoma in 14 (1.41%).

Patients in which neuromonitoring was utilized (group B) were 1041 (2068 nerves at risk), 250 male and 791 female. 895 (85.98%) were submitted to a total thyroidectomy, 14 (1.34%) to a completion total thyroidectomy and 132 (12.68%) to a total thyroidectomy associated to a VI level lymphectomy. In this group diagnosis was benign multinodular goiter in 377 (36.22%) patients, differentiated carcinoma in 330 (31.7%), Hashimoto’s thyroiditis in 208 (19.98%), Graves’ disease in 111 (10.66%), medullary carcinoma in 15 (1.44%).

Groups were homogeneous for characteristics of patients, diagnosis and type of surgery.

We define "transient" an injury in which the motility of the vocal cords recovered within 12 months after surgery.

Parameters were tested using the chi-square test. A logistic regression analysis was fitted using transient and permanent palsy as measurements of outcome. A p value of less than 0.05 was considered to be significant. Statistical analysis was done with SPSS software (SPSS in, Chicago, III).

## Results

In group A 26 unilateral recurrent laryngeal nerve paralysis were observed (2.62%) of which 20 (2.01%) transient and 6 permanent (0.6%). Bilateral palsy was observed in 2 cases (0.2%); in one patient a vocal cord recovered completely three months after surgery. The patient, which has had a completion thyroidectomy, had a nerve unilateral palsy by previous surgery. In the other case, the patient required a tracheostomy and the lesion was permanent. Totally in group A 28 recurrent laryngeal nerve injuries were observed (2.82%), 21 (2.11%) transient and 7 (0.7%) permanent.

In group B 21 unilateral recurrent laryngeal nerve paralysis were observed (2.02%), 15 (1.44%) transient and 6 (0.57%) permanent. In this group of patients a bilateral recurrent laryngeal palsy was observed in two cases (0.19%) requiring a tracheostomy. Totally in group B 23 recurrent laryngeal nerve injuries were observed (2.21%), in 17 cases (1.63%) transient and in 6 (0.58%) permanent. Differences between the two groups were not statistically significative (Table 2).

**Table 2:** Recurrent laryngeal nerve paralysis in the two groups in relation to type of surgery and histology

	**Group A (993 patients) n (%)**	**Group B (1041 patients) n (%)**	**p value**	
Recurrent nerve palsy	28 (2.82%)	23 (2.21%)	0.4604	p > 0.05
• Unilateral	26 (2.62%)	21 (2.02%)	0.4507	p > 0.05
• Bilateral	2 (0.2%)	2 (0.19%)	0.6503	p > 0.05
Transient nerve palsy	21 (2.11%)	17 (1.63%)	0.5232	p > 0.05
• Total thyroidectomy	15 (1.51%)	12 (1.15%)	0.6094	p > 0.05
• Completion thyroidectomy	2** (0.2%)	2 (0.19%)	0.583	p > 0.05
• Total thyroidectomy + lymphectomy	4 (0.4%)	3 (0.29%)	0.6981	p > 0.05
• Multinodular goiter	7 (0.7%)	8 (0.77%)	0.9875	p > 0.05
• Differentiated carcinoma	8** (0.81%)	4 (0.38%)	0.2569	p > 0.05
• Hashimoto’s thyroiditis	2 (0.2%)	4 (0.38%)	0.647	p > 0.05
• Graves’ disease	2 (0.2%)	1 (0.1%)	0.9546	p > 0.05
• Medullary carcinoma	0	0	-	-
Permanent nerve palsy	7 (0.7%)	6 (0.58%)	0.9319	p > 0.05
• Total thyroidectomy	5* (0.5%)	5* (0.48%)	0.8081	p > 0.05
• Completion thyroidectomy	1 (0.1%)	0	0.5792	p > 0.05
• Total thyroidectomy + lymphectomy	1 (0.1%)	1 (0.1%)	0.6082	p > 0.05
• Multinodular goiter	2* (0.2%)	3 (0.29%)	0.9928	p > 0.05
• Differentiated carcinoma	3 (0.3%)	2* (0.19%)	0.8691	p > 0.05
• Hashimoto’s thyroiditis	1 (0.1%)	1 (0.1%)	0.4951	p > 0.05
• Graves’ disease	1 (0.1%)	0	0.974	p > 0.05
• Medullary carcinoma	0	0	-	-

*One patient had bilateral nerve palsy.

**One patient with prior monolateral nerve palsy had controlateral nerve palsy in completion thyroidectomy.

The mean postoperative hospital stay was 2 days in both groups.

In group B we had 21 true positive cases, 1012 true negative, 6 false positive and 2 false negative. Accuracy of neuromonitoring was 99.2%, positive predictive value 77.8%, negative predictive value 99.8%. Sensitivity was 91.3% and specificity 99.4% (Table 3).

**Table 3:** Correlation of neuromonitoring results with postoperative outcomes

	
True positives	21
True negatives	1012
False positives	6
False negatives	2
Accuracy	99.23%
Positive predictive value	77.77%
Negative predictive value	99.8%
Sensitivity	91.3%
Specificity	99.41%

Recurrent laryngeal nerve paralysis in the two groups in relation to type of surgery and histology are reported in Table 2. Differences were not statistically significant.

No complications were attributable to the use of intraoperative neuromonitoring.

## Discussion

The incidence of recurrent laryngeal nerve palsy varies from less than 1% to as high as 20% [26,32,38]. Several factors influence the likelihood of nerve injury, including the underlying disease, the extent of resection, and the experience of the surgeon [16,17,39]; substernal goiters and reoperative thyroid procedures have a higher rate of complications because of scarring and loss of normal tissue planes [32,40-42]. Even experienced surgeons report inadvertent injury to the nerve and persistent palsy in about 1-2% of patients [32,38,39,43]. The reported causes of recurrent laryngeal nerve injury result from damage to the nerve’s anatomic integrity, thermal lesions, excessive nerve skeletization, axon damage caused by excessive strain, edema, hematoma, difficult tracheal intubation, and neuritis [44]; but the actual causes of recurrent laryngeal nerve injury, especially in those with visual integrity, are still not well understood [27]. Several studies have shown that surgeons were not aware of nerve injury during the operation in most instances [27].

Visual identification of recurrent laryngeal nerve during thyroid operations has been associated with lower rates of permanent recurrent laryngeal nerve palsy and is considered the gold standard of recurrent laryngeal nerve treatment by many studies [27]. In 1938 Lahey first dissected the recurrent laryngeal nerve in virtually every case; careful dissection decreased the number of injuries to the recurrent nerves and this approach is accepted by most endocrine surgeons [26]. Nerve identification in certain types of operations may be very difficult: these difficult cases include reoperations, surgery for malignant disease, anatomic variants, and a history of irradiation or inflammation [16,26,45]. Nerve monitoring has been developed to facilitate the identification of the recurrent laryngeal nerve during thyroid surgery [18,23,26,45] and it may provide guidance for the surgeon in such difficult situations [45].

There are a wide variety of techniques for nerve monitoring but the most common method in current use is a special endotracheal tube with electrodes embedded on it that register effects of stimulation in the vocal cords [25,46]. Despite the increasing popularity of this technology, its impact in the avoidance of nerve injury and the reduction of the incidence of postoperative nerve palsy remain doubtful [23,25].

Several non-randomised studies demonstrated that an improved intraoperative recurrent laryngeal nerve identification rate with neuromonitoring resulted in lower palsy rates than in historical patient cohorts with identification alone [19]. The largest non-randomized multicenter trial conducted in Germany with more than 16,000 patients reported that the device could help in decreasing the risk of nerve injury [13,19,46].

A large multicentre study of 4382 patients found a statistically significant reduction in rates of transient and permanent recurrent laryngeal nerve paralysis with the use of intraoperative nerve monitoring in surgery for benign goiter; subgroup analysis of the same study found higher rates of injury when intraoperative nerve monitoring was used for total thyroidectomy [19,25].

On the other side, a large single-centre study of 1000 consecutive nerve at risk found that continuous nerve monitoring offered absolutely no benefit in reducing the risk of nerve injury compared with the adoption of routine nerve identification, with no difference in both the temporary and the permanent nerve injury rates [23,25].

In the study of Dralle [6] intraoperative nerve monitoring did not lower the risk of nerve injury although failure to visually identify the nerve was associated with a higher rate of injury; nerve injury rates were also higher for low-volume hospitals and low-volume surgeons and this fact was confirmed by others [5,23,26,33].

In the meta-analysis of Higgins [47], intraoperative neuromonitoring and identification alone did not demonstrate a statistically significant difference in rates of total, transient, or persistent true vocal fold palsy with adequately-powered sample sizes for all thyroid surgeries, primary surgery, benign disease, and low-risk groups.

Other studies claim that the nerve monitoring is really of value only in high-risk surgery, re-operative cases, or recurrent goiters [25,33], but an impractically large number of patients would be needed because of the rarity of nerve injury that is achieved in specialized centers [23].

In a prospective, randomized study of 1,000 patients (1,000 nerves at risk for each group), the prevalence of transient recurrent laryngeal nerve palsy was significantly lower in the group of patients who had surgery with intraoperative neuromonitoring by 2.9% in high-risk patients and 0.9% in low-risk patients [5,48]. The rate of permanent nerve palsy tended to be lower in operations performed with intraoperative neuromonitoring (by 30%), but the power of this study was not sufficient to validate this difference [5].

In our study the rates of postoperative recurrent laryngeal nerve paralysis did not differ significantly between the two groups. The rate of transient and definitive recurrent laryngeal nerve paralysis were 2.11% and 0.7% respectively in group A (visualization alone) and 1.63% and 0.58% in group B (with neuromonitoring); this slight difference showed no statistical significance. No statistically significant difference in the rate of vocal cord paralysis was found in relation to the most common histological diagnosis. The main weakness with this and other studies on this topic is just the inadequate power to identify a statistical difference and this is a significant limitation. The patients were not randomized which could lead to bias, but the number of patients is very large (2034, 4014 nerves at risk) and the groups are very well matched. To our knowledge, this is the largest mono-institutional study published in the literature to date. Our opinion is that neuromonitoring can help in the nerve identification particularly in difficult situation such as reoperative surgery or anatomic variations but it is very difficult to demonstrate a statistically significant reduction in the incidence of injuries because an extremely large number of patients would be needed. However it must be said that difficult situations are not always predictable preoperatively.

It has been observed that an anatomically intact nerve does not always correlate with normal vocal fold function and that the absence of signal does not necessarily imply nerve dysfunction [33].

In the study of Thomusch [19] a normal intraoperative neuromonitoring signal certainly excluded postoperative vocal cord dysfunction. Indirect stimulation of the recurrent laryngeal nerve via the vagus nerve was a significantly better predictor of postoperative dysfunction than direct stimulation and should be always performed to monitor the axonal excitability and mechanical intactness of the nerve. Direct nerve stimulation is exclusively recommended to detect the recurrent laryngeal nerve anatomy. After indirect nerve stimulation and a normal intraoperative neuromonitoring signal, the surgeon can extend the operation to the contralateral side, being reassured that the nerve on the resected side has a 99.6% chance of being intact [19].

In some cases an absent or abnormal intraoperative neuromonitoring signal failed to predict reliably a postoperative palsy. This failure may be due to problems with the technical device, wrong application or continuous relaxation during the operation with paralysed vocal musculature, resulting in an absent signal. An absent or abnormal signal was a rare phenomenon, with an incidence of 2.7% after indirect recurrent laryngeal nerve stimulation, and in these cases, 29.8-45.9% of the patients had postoperative vocal cord dysfunction [19].

Our experience confirm the high accuracy (99.23%), sensitivity (91.3%) and specificity (99.41%) of this method; positive predictive value was 77.77% and negative predictive value 99.8% confirming the data reported in the literature.

The use of these devices in thyroid surgery seems to be more expensive than the conventional technique. This is probably the major disadvantage [26] and particularly for this reason routine nerve monitoring is no cost-effective [25].

The proponents of routine nerve monitoring claim that its use can still be justified for bilateral surgery stating that if nerve monitoring is undertaken after completion of one side of the procedure and there is evidence of impaired function, then the procedure should be abandoned to avoid the risk of bilateral damage [5,18,19,25,45]. A failed intraoperative neuromonitoring stimulation of the recurrent laryngeal nerve after resection of the first lobe is specific enough to reconsider the surgical strategy in patients with bilateral thyroid disease to surely prevent bilateral recurrent laryngeal palsy [49].

Neuromonitoring provides comfort during thyroidectomy for identification, dissection, and control of recurrent laryngeal nerves; it can reduce the stress associated with nerve dissection when a surgeon operates on a patient with a challenging anatomy or in cases of extensive surgery [7] but the main benefit remains its ability to guide the surgeon in cases of anatomical variations [45]. However the use of neuromonitoring in only selected cases does not allow adequate familiarity with the device and can lead to pitfalls.

In our experience, the use of this technique has decreased the stress of the surgeon, facilitated the identification of the nerve and given greater safety in the prosecution of the most difficult operations. These effects, however, are very difficult to quantify. Recurrent laryngeal nerve paresis occurrence diminishes the quality of life for a few weeks to months after thyroid surgery; so any improvement in this field is welcome [5]. Recurrent laryngeal nerve monitoring also allows for nerve function documentation before and after thyroid resection (by printing the electromyographic signal of evoked potentials), which is of great importance in an increasing number of litigation [5].

## Conclusions

Visual nerve identification remains the gold standard of recurrent laryngeal nerve management in thyroid surgery. The technique of intraoperative neuromonitoring is safe, effective, and reliable in excluding postoperative recurrent laryngeal nerve palsy. Neuromonitoring helps to identify the recurrent laryngeal nerve, in particular in difficult cases, but it did not decrease recurrent laryngeal nerve injuries compared with visualization alone in this study. Future studies are warranted to evaluate the benefit of intraoperative neuromonitoring in thyroidectomy, especially in conditions in which the recurrent laryngeal nerve is at high risk of injury.

## Competing interests

The authors declare that they have no competing interests.

## Authors’ contributions

PGC conceived of the study and drafted the manuscript. GP carried out part of the operations and helped to draft the manuscript. FM participated in the design of the study and performed the statistical analysis. MRP participated in the design of the study and helped to draft the manuscript. LG worked on follow-up of patients and helped to draft the manuscript. RD participated in the design of the study and helped to draft the manuscript. AN participated in the design and coordination of the study and helped to draft the manuscript. All authors read and approved the final manuscript.
